# Untargeted metabolomics unravels functionalities of phosphorylation sites in *Saccharomyces cerevisiae*

**DOI:** 10.1186/s12918-016-0350-8

**Published:** 2016-11-15

**Authors:** Zrinka Raguz Nakic, Gerhard Seisenbacher, Francesc Posas, Uwe Sauer

**Affiliations:** 1 Institute of Molecular Systems Biology, ETH Zürich, Auguste-Piccard-Hof 1, Zürich, Switzerland; 2 PhD Program on Systems Biology, Life Science Zürich, Zürich, Switzerland; 3 Cell signaling Research Group, Departament de Ciències Experimentals i de la Salut, Universitat Pompeu Fabra, Barcelona, Spain

**Keywords:** Phosphorylation, Metabolism, Post-translational modification, TOR signaling, Osmotic stress

## Abstract

**Background:**

Coordinated through a complex network of kinases and phosphatases, protein phosphorylation regulates essentially all cellular processes in eukaryotes. Recent advances in proteomics enable detection of thousands of phosphorylation sites (phosphosites) in single experiments. However, functionality of the vast majority of these sites remains unclear and we lack suitable approaches to evaluate functional relevance at a pace that matches their detection.

**Results:**

Here, we assess functionality of 26 phosphosites by introducing phosphodeletion and phosphomimic mutations in 25 metabolic enzymes and regulators from the TOR and HOG signaling pathway in *Saccharomyces cerevisiae* by phenotypic analysis and untargeted metabolomics. We show that metabolomics largely outperforms growth analysis and recovers 10 out of the 13 previously characterized phosphosites and suggests functionality for several novel sites, including S79 on the TOR regulatory protein Tip41. We analyze metabolic profiles to identify consequences underlying regulatory phosphorylation events and detecting glycerol metabolism to have a so far unknown influence on arginine metabolism via phosphoregulation of the glycerol dehydrogenases. Further, we also find S508 in the MAPKK Pbs2 as a potential link for cross-talking between HOG signaling and the cell wall integrity pathway.

**Conclusions:**

We demonstrate that metabolic profiles can be exploited for gaining insight into regulatory consequences and biological roles of phosphosites. Altogether, untargeted metabolomics is a fast, sensitive and informative approach appropriate for future large-scale functional analyses of phosphosites.

**Electronic supplementary material:**

The online version of this article (doi:10.1186/s12918-016-0350-8) contains supplementary material, which is available to authorized users.

## Background

Adaptation to changes in the environment is crucial for the survival of any organism and adjustment of protein levels and activities is pivotal for this process. Long-term adaptations via (post-)transcriptional regulation or irreversible protein modification typically affect protein abundance. Fast adaptations are mediated by allosteric regulators and a chemically diverse group of covalent post-translational modifications (PTMs). In eukaryotes, phosphorylation stands out among PTMs for the broad scope of regulation and the complexity of its network with over 160 known kinases and phosphatases in *Saccharomyces cerevisiae* [1]. Recent advances in proteomics allow for deep interrogation of phosphoproteomes, leading to large catalogues of phosphorylation sites (phosphosites) in different species [2–4], suggesting that as many as 45% of all proteins in eukaryotes may be phosphorylated [5]. While the number of mapped phosphosites is ever increasing, we still rarely know whether phosphorylation events have a functional role. A substantial fraction of all phosphosites was suggested to be non-functional, resulting from stochastic off-target kinase activity [6, 7]. Typically, only a handful of all observed phosphosites in any organism has been functionally validated and characterized [5, 8] because the success in phosphoproteomics in detecting phosphosites has largely outpaced the performance and throughput of the available approaches for analysis of phosphosites functionality.

Present workflows for functional analysis of phosphosites typically start with phosphoproteomics to generate catalogues of modified amino acids. Computational approaches then distinguish between relevant and non-functional phosphosites on the basis of different criteria including kinase recognition motives [9, 10], conservation patterns of phosphosites across species [6, 7], correlations of changes in phosphorylation state with metabolic flux [11], or temporal patterns of phosphosite appearances upon disturbance [12]. After prioritization of interesting phosphosites, their functionality remains to be determined in laborious follow-up experiments. Typically, these experiments take advantage of site specific phosphosite mutants and include a combination of phosphorylation assays, growth and viability assays, localization studies, expression studies and enzymatic in vitro assays [13–20]. Targeted measurements of intracellular metabolites have been used in some cases to investigate phosphoregulation of metabolism [11, 16, 20]. All these methods have been powerful in elucidating the importance of single phosphosites. However, they are not suitable for high throughput analysis as the readout of most approaches is specific to the investigated protein and would imply tedious laboratory work to make them applicable in larger scale. Although growth and viability assays allow high throughput, their sensitivity for assessing functionality is unclear. Given that phosphorylation often only fine-tunes the activity of a protein, interfering with phosphorylation might not necessarily penetrate to the phenotypic level.

The recent developments in genome editing render the generation of large numbers of mutants with modified phosphosites for functional analysis feasible [21–24]. Hence, the limitation shifts to a suitable high throughput approach for subsequent mutant analysis, in particular when phenotypes are more subtle, as can be expected for most point mutations. In principle the various omics technologies would be applicable, but in practice they are typically too laborious or expensive. A notable exception is mass spectrometry-based metabolomics. In particular, it offers the potential to identify small differences in metabolite profiles well before a change of the phenotype is observed [25, 26]. Since many of the phosphoproteins listed in phosphosite databases are part of the metabolic network, metabolomics is a promising approach for probing their functionality.

Here we investigate the potential of untargeted metabolomics for higher throughput identification and characterization of phosphosite functionality. We introduced pointmutations into 26 phosphosites from 25 proteins in *S. cerevisiae*, several of which were known to be functionally regulated by phosphorylation. The proteins included metabolic enzymes and regulatory proteins implicated in the TOR or HOG signaling, two pathways closely related to metabolism [27–29]. The phosphoresidues were mutated to amino acids that mimic a constitutively dephosphorylated and, for a selected subset, phosphorylated state, resulting in a total of 32 phosphomutants. Applying untargeted metabolomics, we retrieved most previously characterized phosphoproteins as functionally relevant and suggest functionality for additional phosphosites. We also showed that untargeted metabolomics aids in unveiling the biological roles of functional phosphosites in regulating metabolic pathways. Our results demonstrate that untargeted metabolomics is a highly sensitive method for high throughput detection of functionalities of phosphosites.

## Results

### Phenotypic analysis reveals functionalities of strong-impact phosphosites

For the purpose of defining a set of phosphomutants for phenotypic and metabolic analysis, we selected single phosphosites or multiple phosphosites in close proximity (hereafter jointly termed as phosphosites) on metabolic enzymes or proteins involved in TOR or HOG signaling. The 26 chosen phosphosites were located on 25 proteins that represent different functional classes, such as metabolic enzymes, kinases or transcription factors (Table 1 and Additional file 1). The two signaling pathways were selected for their metabolic function, rendering them amenable for a metabolomics approach. TOR signaling controls the cellular response to nitrogen availability, having a substantial impact on the state of metabolism when metabolizing nitrogen sources of different quality [27, 29]. The main role of the HOG signaling pathway is sensing of extracellular hyperosmolarity and eliciting a stress response, which in *S. cerevisiae* primarily consists of a metabolic adaptation to increase the concentration of the osmolyte glycerol together with cell cycle and transcriptional modulation [28]. The total set of the 26 phosphosites contained 13 functionally characterized and 13 uncharacterized phosphosites. We classified phosphosites as characterized when a phosphosite mutant has been reported to have an effect in any type of functional analysis. Most uncharacterized phosphosites were selected based on differential phosphorylation data from phosphoproteomics studies in either different growth conditions or upon kinase deletion [4, 11, 30] (Additional file 1). The chosen phosphosites were mutated to alanine whenever serine or threonine was the phosphoresidue, or phenylalanine if tyrosine was the phosphoresidue, thereby abolishing phosphorylation of the target amino acid (Table 1). For six out of the 26 phosphosites the residues were additionally mutated to glutamic acid to mimic a constitutively phosphorylated state of the protein, resulting in a total of 32 mutants. Throughout this study, mutants with abolished phosphorylation are referred to as “OUT” mutants, while phosphomimic mutants are referred to as “IN” mutants. Up to three independent mutant replicates per phosphosite were generated (Additional file 1). Phosphomutant replicates with broad metabolic effects exhibited highly similar metabolic profiles (Additional file 2: Figure S1), suggesting that genetic manipulations were precise.

**Table 1:** Phosphoprotein mutant set

Standard Name	Systematic name	Position of mutation within protein	Mutation type	Mutant nomenclature	Protein type	Pathways contribution	Evidence type
Crz1	YNL027W	409, 410, 423, 427, 429	p-deletion	Crz1 OUT	TF	HOG pathway	ch [38, 61]
Cys3	YAL012W	39, 40	p-deletion	Cys3 OUT	Enzyme	metabolic enzyme	dif [11, 30]
Gln3	YER040W	469, 471, 473	p-deletion	Gln3 site 1 OUT	TF	TOR signaling	dif [4, 11]
Gln3	YER040W	469	p-deletion	Gln3 site 2 OUT	TF	TOR signaling	dif [4, 11]
Gpd1	YDL022W	24, 27	p-deletion	Gpd1 OUT	Enzyme	metabolic enzyme	ch [11, 20]
Gpd2	YOL059W	72, 75	p-deletion	Gpd2 OUT	Enzyme	metabolic enzyme	ch [20]
Gut1	YHL032C	96	p-deletion	Gut1 OUT	Enzyme	metabolic enzyme	dif [11]
Hog1	YLR113W	174, 176	p-deletion	Hog1 OUT	Kinase	HOG pathway	ch [31, 62]
Lys20	YDL182W	395, 396	p-deletion	Lys20 OUT	Enzyme	metabolic enzyme	ch [4, 11, 26]
Nbp2	YDR162C	194, 196	p-deletion	Nbp2 OUT	Adapter	HOG pathway	dif [4, 30]
Nbp2	YDR162C	194, 196	p-mimic	Nbp2 IN	Adapter	HOG pathway	dif [4, 30]
Pbs2	YJL128C	508	p-deletion	Pbs2 OUT	Kinase	HOG pathway	ch [15]
Pbs2	YJL128C	508	p-mimic	Pbs2 IN	Kinase	HOG pathway	ch [15]
Pda1	YER178W	313	p-deletion	Pda1 OUT	Enzyme	metabolic enzyme	ch [63]
Pda1	YER178W	313	p-mimic	Pda1 IN	Enzyme	metabolic enzyme	ch [63]
Pfk1	YGR240C	895	p-deletion	Pfk1 OUT	Enzyme	metabolic enzyme	dif [4, 11]
Pfk2	YMR205C	163	p-deletion	Pfk2 OUT	Enzyme	metabolic enzyme	dif^a^ [4, 11]
Pfk2	YMR205C	163	p-mimic	Pfk2 IN	Enzyme	metabolic enzyme	dif^a^ [4, 11]
Put3	YKL015W	788	p-deletion	Put3 OUT	TF	TOR signaling	ch [18]
Rim15	YFL033C	1061	p-deletion	Rim15 OUT	Kinase	TOR signaling	ch [64]
Rim15	YFL033C	1061	p-mimic	Rim15 IN	Kinase	TOR signaling	ch [64]
Sko1	YNL167C	108, 113, 126	p-deletion	Sko1 OUT	TF	HOG pathway	ch [65]
Smp1	YBR182C	348, 357, 365, 376	p-deletion	Smp1 OUT	TF	HOG pathway	ch [66]
Ste50	YCL032W	202	p-deletion	Ste50 OUT	Adapter	HOG pathway	ch [37]
Str3	YGL184C	456	p-deletion	Str3 OUT	Enzyme	metabolic enzyme	htp [30]
Tco89	YPL180W	287, 288, 290	p-deletion	Tco89 OUT	Regulator	TOR signaling	dif [4]
Thr4	YCR053W	408	p-deletion	Thr4 OUT	Enzyme	metabolic enzyme	dif [11]
Tip41	YPR040W	79	p-deletion	Tip41 OUT	Regulator	TOR signaling	dif [4]
Tkl1	YPR074C	335	p-deletion	Tkl1 OUT	Enzyme	metabolic enzyme	htp [30]
Tkl1	YPR074C	335	p-mimic	Tkl1 IN	Enzyme	metabolic enzyme	htp [30]
Yap4	YOR028C	192, 196	p-deletion	Yap4 OUT	TF	HOG pathway	ch [19]
Zwf1	YNL241C	183, 188	p-deletion	Zwf1 OUT	Enzyme	metabolic enzyme	dif [11]

*TF* transcription factor, *ch* previously characterized phosphosite, *dif* phosphosite shown in previous phosphoproteomics studies to change in degree of phosphorylation in different conditions, *htp* phosphosite detected in high throughput phosphoproteomics studies, no data on differential phosphorylation

^a^The previously shown functionality of phosphorylation of Pfk2 S163 is potentially a result of increased expression from the plasmid used in that study (Additional file 2: Figure S6)

**Fig. 1:**
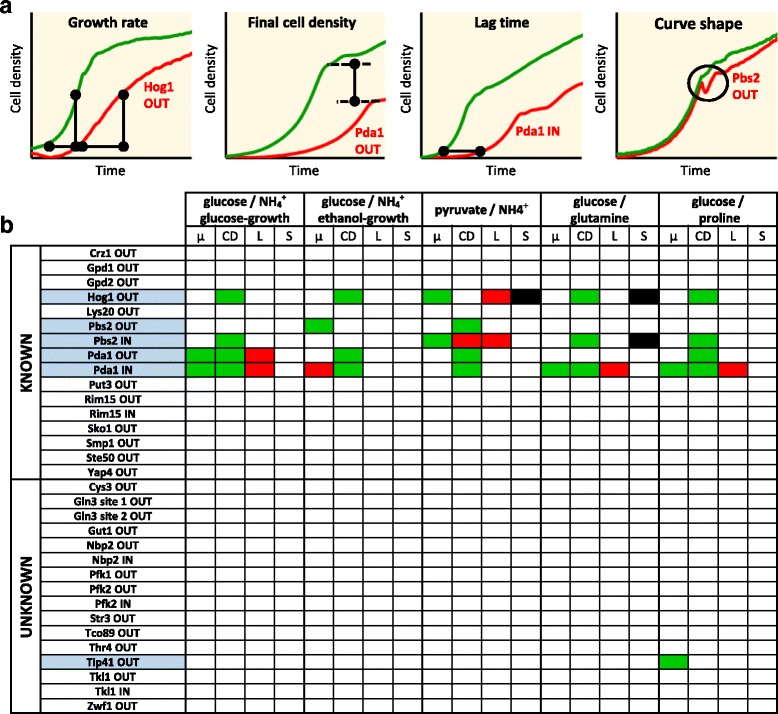
Growth phenotype of *S. cerevisiae* phosphomutants. **a** Example curves for analyzed growth traits of wild-type (*green*) and mutant (*red*). **b** Growth phenotype in different growth conditions. Results that differ significantly from the wild-type are highlighted. *Red* and *green boxes* indicate higher or lower results for the respective growth trait of the mutant compared to the wild-type. *Black boxes* indicate a general difference in shape. Mutants with a growth phenotype are highlighted in *blue*. Abbreviations: μ: growth rate. CD: maximum cell density in one growth phase. L: lag time. S: abnormalities in shape of growth curve

To identify condition-specific regulation, the 32 phosphomutants were grown on four combinations of carbon and nitrogen sources that require different activities of the mutated proteins, such as glycolytic or gluconeogenic fluxes, nitrogen sources of different quality, and low osmotic stress on 0.22 M sodium pyruvate (Table 2). We quantified growth rates and maximum cell density and qualitatively assessed lag times and other abnormalities in the growth curves of microtiter plate cultures (Fig. 1a and Additional file 3). Six mutants in four different phosphosites exhibited an aberrant phenotype under at least one condition (Fig. 1b). Three of these phosphosites were already previously characterized, but functionality of the Tip41 phosphosite was hitherto unknown. The phosphomutants of Pda1 and Pbs2 showed varying effects under most conditions, whereby the IN mutant was more strongly affected in both proteins. The IN mutant of the kinase Pbs2 showed a very similar behavior as its target Hog1 OUT, supporting the hypothesis that phosphorylation of Pbs2 S508 inactivates the protein, while in Hog1, phosphorylation of S174 and T176 in the activation loop activates this kinase [15, 31, 32]. Overall, we retrieved three out of the 13 known phosphoregulated proteins under the tested conditions. Thus, growth phenotypes provide strong evidence for phosphosite functionality but would require testing of many conditions.

**Table 2:** Carbon and nitrogen sources of media for growth and metabolomics experiments

Condition	Carbon sources	Nitrogen sources	Mode of glycolysis	Quality of nitrogen source
Glucose/NH_4_^+^	Glucose	NH_4_^+^	glycolytic	good
Pyruvate/NH_4_^+^	Pyruvate	NH_4_^+^	gluconeogenic	good
Glucose/Glutamine	Glucose, Glutamine	Glutamine	glycolytic	good
Glucose/Proline	Glucose, Proline	Proline	glycolytic	poor

### Untargeted metabolomics reveals changes in phenotypically silent phosphomutants

Next, we tested whether metabolic functionality of phosphosites whose genetic perturbation does not propagate to physiological phenotypes could be identified by untargeted metabolomics. Intracellular metabolites were extracted with hot ethanol from exponentially growing cultures in the four before used conditions using 96-well cultivation. The extracts were injected into a Time Of Flight mass spectrometer (TOF-MS) and metabolic profiles in the m/z range of 50 to 1000 Dalton were recorded [33]. Across all conditions, 122–259 ions were annotated to 183–342 metabolites using a genome-wide reconstruction model of *S. cerevisiae* [34] (Additional file 2: Table S1). The data was processed to remove intensity drifts during measurements and an OD-specific fold change was calculated for every ion of each metabolic profile. The fold changes of the replicates of one mutant were compared to the fold changes of all other samples using a 2-sample t-test and the median log2 fold change over all replicates was determined. Ions with a corrected *p*-value < 10^−3^ and a |log2 fold change| > 0.3785 were considered as changing significantly. The selected log2 fold change cutoff corresponds to a 30% change and represents the 97.5% quantile across the fold changes of all ions from the wild-type dataset (Additional file 2: Figure S2A).

**Fig. 2:**
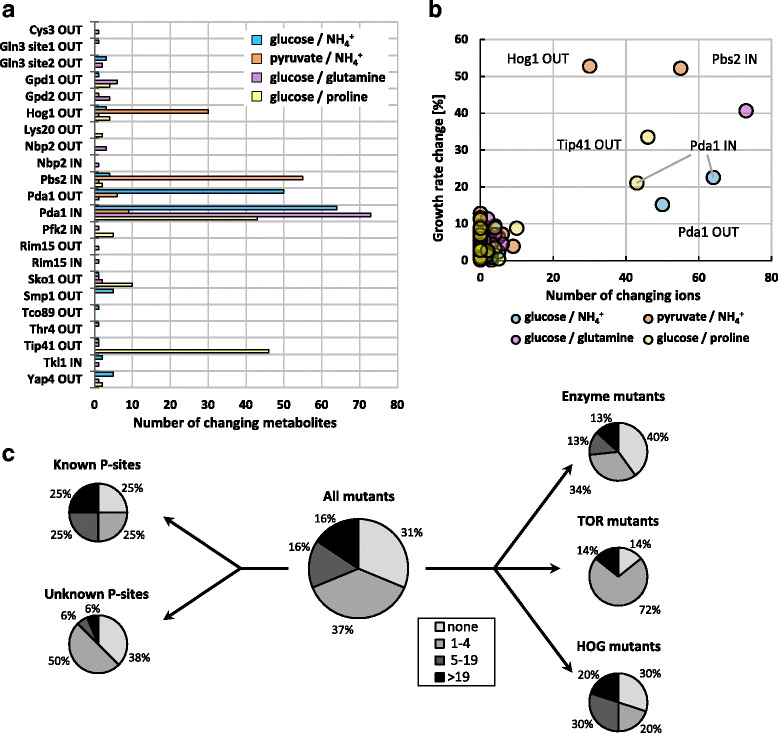
Overview on changing ions in metabolic profiles. **a** Number of changing ions of each mutant and condition. Only mutants with significantly changing ions are depicted (|log2 fold change| > 0.3785 and *p*-value < 10^−3^). **b** Relation between number of changing ions and mutant growth rate. **c** Distribution of changing ions in different mutant subclasses with no (none), little (1–4), intermediate (5–19) or many (>19) changing ions

Typically, metabolite concentrations changed less than two-fold in the phosphomutants (Additional file 2: Figure S2B) and the majority of changes were in arginine, proline, pyrimidine, purine or lysine metabolism (Additional file 2: Figure S4). While 31% of the mutants exhibited no significant (*p*-value < 10^−3^, |log2 fold change| > 0.3785) metabolic response, most exhibited changes in some metabolites, and five of the six mutants with physiological phenotypes (Hog1 OUT, Pbs2 IN, Pda1 OUT, Pda1 IN and Tip41 OUT) showed changes in more than 15 metabolites in at least one condition (Fig. 2a). In many cases these broadly changing metabolic profiles were presumably the consequence of altered growth rates (Fig. 2b). Generally, phosphomutants with previously characterized phosphosites featured more changes than the unknown subset, suggesting that the latter contains more nonfunctional sites (Fig. 2c). Metabolic profiles of phosphosites with strong metabolic impact often showed a tendency towards condition-specific effects, demonstrating the regulatory role of phosphosites under particular conditions. Hog1 OUT for example showed broad effects only when grown on pyruvate as the carbon source, presumably because of the elevated osmotic stress in this condition, and Tip41 showed almost exclusively effects when growing on proline as sole nitrogen source (Fig. 2a). Overall, our data suggest that metabolomics can detect subtle consequences of deregulated phosphorylation that is without detectable growth phenotypes.

### Metabolic profile analysis indicates phosphosite functionality

**Fig. 3:**
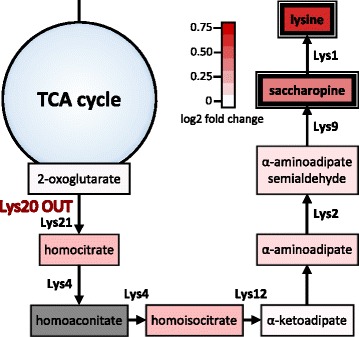
Metabolite level changes in lysine biosynthesis of the Lys20 OUT mutant growing on glucose/proline. *Coloring* indicates the strength and direction of the fold changes. Significant fold changes are highlighted with a *bold frame* (*p*-value < 10^−3^, absolute log2 fold change > 0.3785). Metabolites in *grey* were not detected

While we did not detect changes in direct reactants of the 15 phospho-mutated enzymes after applying a strict cutoff, we found metabolite changes for 12 mutants to be enriched in specific pathways, suggesting phosphorylation to be functionally important for these pathways (Additional file 4). For example, the OUT mutant of Yap4, a transcription factor implicated in stress responses, showed changes enriched in purine metabolism when growing on glucose/glutamine (*p*-value 0.0007). Metabolic effects of the Pda1 phosphomutants predominantly affected arginine and proline metabolism (*p*-value 0.005 for Pda1 IN and *p*-value 0.0004 for Pda1 OUT respectively in glucose/NH_4_^+^, *p*-value 0.0003 for Pda1 IN in glucose/glutamine). A particularly clear case was Lys20, one of two homocitrate synthase isoenzymes that catalyze the conversion of oxoglutarate to homocitrate, the first step in lysine biosynthesis. Lysine metabolism was found to be exclusively enriched (*p*-value 0.006) in Lys20 OUT when growing on glucose/proline. While lysine biosynthesis is known to be subject to transcriptional and allosteric control, it was recently shown that Lys20 activity is also regulated by phosphorylation [26, 35]. Metabolite responses in Lys20 OUT were observed only during growth on the poor nitrogen source proline and significant (*p*-value < 10^−3^, |log2 fold change| > 0.3785) changes were restricted to the final pathway product lysine and its precursor saccharopine (Fig. 3). Consistent with previous results [26], increased lysine levels in Lys20 OUT suggest phosphorylation at position S395/T396 to deactivate the enzyme. Since lysine biosynthesis flux is rather low during slow growth on proline, our data indicates that the pathway capacity is down-regulated by phosphorylation in this condition.

To identify more global impact of phosphosites on metabolism, we used two correlation approaches to analyze the metabolic profiles. In the first, we correlated log2 fold changes between all 496 possible mutant-pairs for each condition. The correlation coefficients of all mutant-pairs showed a normal distribution around 0, with a mean of 0.014 (Additional file 2: Figure S5A). The four correlation coefficients from each mutant-pair were compared against the distribution of all derived coefficients to identify significantly correlation pairs. Five mutant-pairs correlated significantly across the four conditions (*p*-value < 0.01), suggesting these phosphosites to exhibit similar effects on metabolism (Additional file 5). Two of these five pairs were mutant variants of the same protein (Gln3 site1 OUT – Gln3 site2 OUT and Rim15 OUT – Rim15 IN), emphasizing similar functionality of the introduced mutations. Rim15 OUT, implicated in cell cycle regulation in response to nutrient, and Pda1 IN were negatively correlated in all conditions, whereby the strongest opposing effects were largely in amino acid metabolism. The two other cases were Pfk2 OUT associating with Ste50 OUT as well as Yap4 OUT.

In the second correlation, we quantified the similarity of each mutant with itself across all four conditions to identify phosphosite functionalities based on subtle but consistent changes in metabolites across conditions. The correlation coefficients of all condition-pairs showed an average above zero, emphasizing that our phosphomutant set contains mutants with condition-independent metabolic effects (Additional file 2: Figure S5B). Out of the eight phosphomutants that were recovered in this analysis, we already described strong metabolic changes predominantly under one condition for three mutants (Pda1 OUT, Hog1 OUT and Pbs2 IN, see Additional file 6). The calculated correlation coefficients were particularly weak for Hog1 OUT and Pbs2 IN. We suspect that despite the predominant effects in specific conditions, this weak similarity is driven by underlying subtle, but condition-independent, metabolic changes. One such example in Pbs2 IN is the slight depletion of glycerol across conditions, emphasizing the role of Pbs2 in controlling glycerol accumulation via the HOG pathway even under non-stress conditions [36]. Additionally, the Gpd2 OUT, Gut1 OUT, Sko1 OUT and Lys20 OUT mutants associated with themselves across conditions. These mutants are cases without strong effects under any tested condition, but with subtle condition-independent changes, which suggest a possible fine-tuning function of the phosphosites. The respective metabolites driving condition-independent correlations were diverse, e.g. affecting nucleotide metabolism in Gut1 OUT and lysine in Lys20 OUT. As expected, we also recovered Pda1 IN that has effects throughout all conditions.

**Fig. 4:**
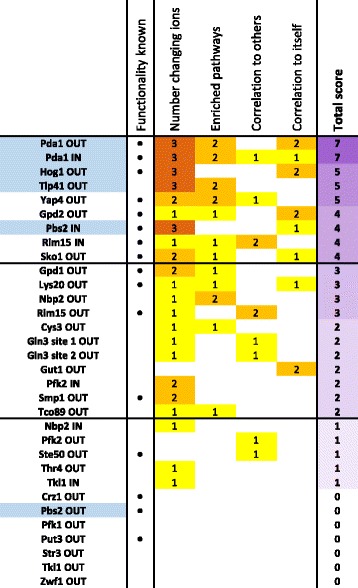
Phosphomutant functionality scoring based on metabolic profiles. The number of changing ions was scored between 0 and 3 according to the following scheme: 0 for no change; 1 for 1–4 changes; 2 for 5–19 changes; 3 for > 19 changes. The remaining three criteria were scored between 0 and 2 based to on the strongest *p*-value: 0 for *p*-value > 10^−2^; 1 for *p*-value < 10^−2^; 2 for *p*-value < 10^−3^. Mutants exhibiting a growth phenotype in the growth analysis are highlighted in *blue*

To systematically interpret the evidence for functionality of a given mutated phosphosite, we ranked the functionality evidence based on a score that reflects the number of changing ions, enriched pathways, correlation with other mutants, and correlation with itself for each phosphosite (Fig. 4). The number of changing ions was scored between 0 and 3. The changes were classified as either little (1–4 ions), affecting equal or less ions than in the wild-type, where 4.75 ions were changing on average, intermediate (5–19 ions), or many (>19 ions) changes, affecting a broad part of metabolism with changes in > 10% of all averagely detected ions. The growth condition with the most changes was considered for scoring. The three other aspects were scored between 0 and 2 according to *p*-values. A score was attributed based on the most significant *p*-value obtained in any growth condition. A *p*-value of at least 10^−2^ was scored with a 1, while a *p*-value below 10^−3^ was scored with a 2. Supporting our approach, most of the already characterized phosphosites scored very high (Fig. 4). The three characterized cases with low scores can be explained by incomplete mutation of all relevant phosphosites (e.g. Ste50) or lack of protein relevance under the tested condition (e.g. Crz1 and Put3) [18, 37, 38]. Our data suggests that the reported phosphoregulation of Pfk2 at position S163 [11] resulted, at least in part, from a non-endogenous increase of Pfk2 levels from the employed plasmid construct (Additional file 2: Figure S6). Since we cannot exclude that the reported phenotype of this Pfk2 variant originated from increased protein abundance, we did not consider Pfk2 S163 as a known functional phosphosite and found only weak functionality evidence in the Pfk2 IN mutant. Overall, the metabolomics data identified most of the already characterized phosphosites as functional with a total score of 3 or higher, one novel regulatory phosphosite with high confidence (Tip41 S79), and several other novel functional phosphosites with lower confidence. The much higher number of phosphosite functionality identification by metabolomics compared to growth phenotype analysis underlines the value of metabolomics in assessing at least metabolic functionality. In the next sections, we exploit the metabolomics data to hypothesize specific functions for phosphosites in the glycerol dehydrogenases Gpd1 and Gpd2, and the MAK kinase Pbs2.

### Phosphorylation of Pbs2 at S508 negatively regulates Hog1 activity

**Fig. 5:**
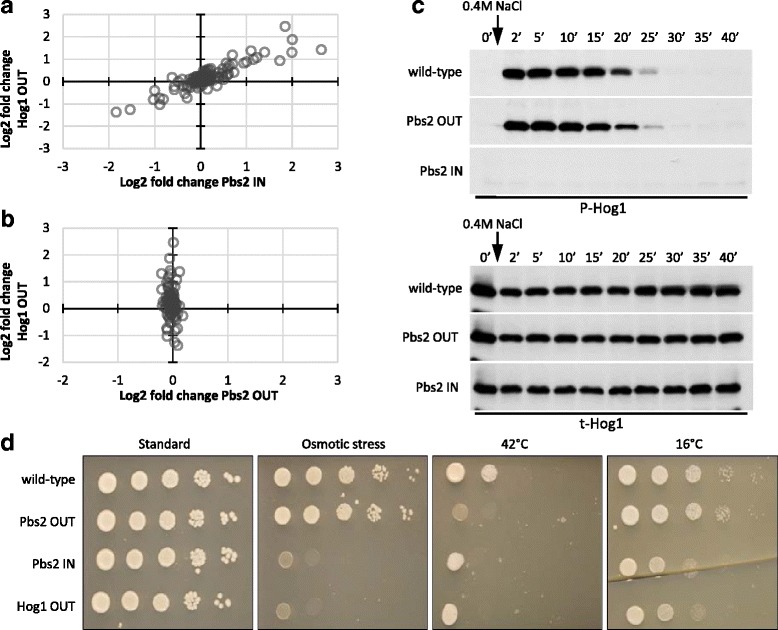
Metabolic profiles, HOG signaling activation and phenotype of Pbs2 phosphomutants. **a** Log2 fold changes of all ions corresponding to deprotonated metabolites of Hog OUT compared to Pbs2 IN in pyruvate/NH_4_^+^ media. **b** Log2 fold changes of all ions corresponding to deprotonated metabolites of Hog OUT compared to Pbs2 OUT in pyruvate/NH_4_^+^ media. **c** Time course Western blot of Hog1 activity in wild-type and Pbs2 phosphomutants, measured as phosphorylated Hog1 (P-Hog1) upon osmotic stress. Total Hog1 (t-Hog1) was measured as control. **d** Phenotype analysis upon osmotic stress and exposure to different temperatures. Spotting assay of Pbs2 phosphomutants and Hog1 OUT in synthetic complete (SC)-glucose, SC-glucose supplemented with 0.5 M NaCl, SC-glucose at 42 °C and SC-glucose at 16 °C. The assay was done three times, the results of one exemplary experiment are shown

Phosphorylation at position S514 and T518 activates the MAPK kinase Pbs2, which in turn activates the Hog1 kinase in response to osmotic stress [28, 39] (Additional file 2: Figure S7). Besides these two well-studies phosphosites, the here investigated S508 phosphosite was suggested to be important for the inactivation of Pbs2 after adaptation to osmotic stress [15]. Our approach scored both Pbs2 IN and its target Hog1 OUT as regulated by phosphorylation (Fig. 4). The strongest metabolic responses of both mutants occurred during growth on pyruvate, presumably due to the osmotic stress of increased sodium concentration in this medium. The striking similarity of the Hog1 OUT and Pbs2 IN profiles and the very different Pbs2 OUT profiles strongly support the hypothesis that phosphorylation at position S508 deactivates Pbs2, which causes reduced Hog1 activity (Fig. 5a and b).

We further investigated the presumably inactivating role of S508 by exposing the Pbs2 mutants to elevated concentrations of NaCl and monitored HOG pathway activity by following Hog1 phosphorylation over time (Fig. 5c). Consistent with the above reasoning, Pbs2 IN failed to phosphorylate and activate Hog1 upon osmotic stress. However, no delay in inactivation of Hog1 was observed for Pbs2 OUT compared to wild-type dynamics, suggesting that deactivation of Pbs2 was not impaired in the Pbs2 OUT mutant. Since the HOG signaling pathway is known to cross-talk with other signaling pathways, we wondered whether phosphorylation of S508 had other physiological roles besides the deactivation of Pbs2 after adaptation to osmotic stress. One such interconnected pathway is the cell wall integrity pathway [40, 41]. To test a link of Pbs2 S508 phosphorylation to cell wall integrity, we grew the mutants at different temperatures to impose different cell wall stress levels. We found that growth of Pbs2 OUT was exclusively impaired at elevated temperatures, while Pbs2 IN and Hog1 OUT showed growth defect most strongly when growing at 16 °C (Fig. 5d). A change in cell wall stress leading to conditional phenotypes of the Pbs2 phosphomutants suggests that phosphorylation of S508 is important for proper activation of HOG signaling under specific cell wall stress conditions and plays a critical role in the cross-talk between the two signaling pathways.

### Phosphorylation of Gpd1 and Gpd2 influences arginine metabolism

**Fig. 6:**
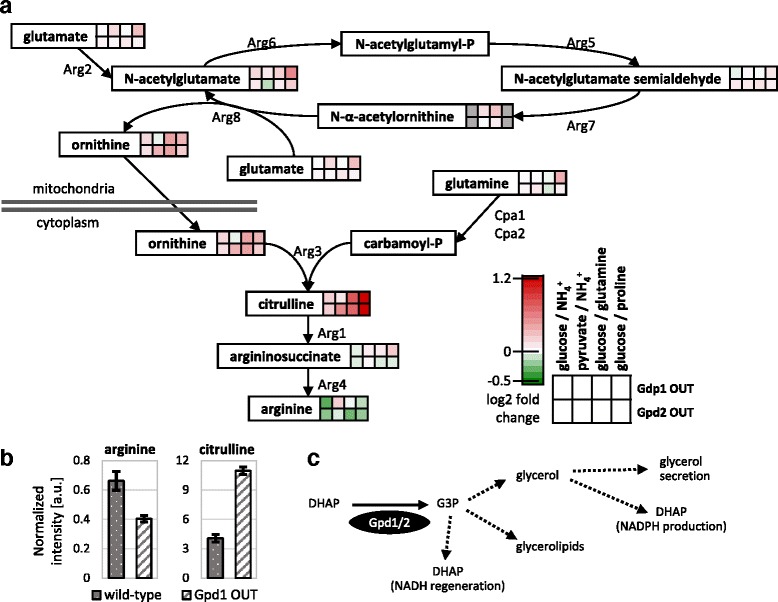
Metabolite responses in arginine biosynthesis of the Gpd1 OUT and Gpd2 OUT mutants. **a** Metabolite response in arginine biosynthesis under all tested conditions. *Coloring* indicates strength and direction of fold changes. Metabolites in *grey* were not detected in the respective conditions. Metabolites without color boxes were not detected. **b** Quantification of arginine and citrulline levels by LC-MS/MS for Gpd1 OUT and wild-type in glucose/NH_4_^+^ medium. **c** Possible metabolic routes for G3P

The glycerol dehydrogenases Gpd1 and Gpd2 catalyze the conversion of dihydroxyacetone phosphate to glycerol-3-phosphate, the first step of glycerol synthesis. Although Gpd1 and Gpd2 have distinct roles, they can at least partially compensate for each other [42, 43]. In addition to transcriptional regulation, both enzymes are subject to phosphoregulation [11, 20]. In our screen, both glycerol dehydrogenases were detected as regulated phosphoproteins, with Gpd2 OUT ranking slightly higher in the metabolic profile analysis than Gpd1 OUT (Fig. 4). Although we did not detect changes in the direct reactants of either of the mutated isoenzymes, both OUT mutants exhibited specific metabolic responses throughout most conditions with an enrichment in arginine and proline metabolism (*p*-value 0.002), in particular when growing on proline as nitrogen source (Additional file 4). Unexpectedly, we detected the strongest changes in citrulline and ornithine, along with some less pronounced changes in arginine that we confirmed by targeted LC-MS/MS measurements (Fig. 6a, b). The striking correlation of Gpd2 OUT with itself across all conditions demonstrates that this phosphoregulation is not specific to the proline condition (Additional file 6).

How glycerol metabolism influences arginine metabolism remains unclear. The OUT mutants have more active glycerol dehydrogenases and hence generate more G3P than the wild-type [11, 20], but neither of the known routes from G3P connects to arginine metabolism (Fig. 6c). Although the observed metabolite changes suggest a possible blockage at Arg1 or Arg4 in the glycerol dehydrogenases phosphomutants (Fig. 6a), the influence is more likely to occur indirectly via a regulatory interaction. Previously, transcriptional and translational regulation of arginine metabolism has been suggested to be implicated in adaptation to osmotic stress [44]. In our study, Pbs2 IN and Hog1 OUT, which have impaired HOG signaling and cannot activate Gpd1 by dephosphorylation, showed strong changes mostly in arginine but also citrulline upon osmotic stress (log2 fold change arginine of 1.845 and 2.463 for Pbs2 IN and Hog1 OUT respectively, see Additional files 7 and 8). These metabolites change in opposite directions compared to the Gdp1 OUT and Gpd2 OUT mutants where arginine is decreased while citrulline is increased, supporting a phosphorylation-dependent influence of the glycerol dehydrogenases on arginine metabolism.

## Discussion

The large networks of kinases and phosphatases represent possibly the most complex post-translational regulation system and play key roles in essentially all cellular processes. Given the rapidly growing collection of reported phosphosites, a major limitation is assessing phosphosite functionality and generating hypotheses on their precise roles, at a scale and speed that matches the high-throughput of modern mapping methods [2, 3, 5, 8, 12, 45]. Here we demonstrate that untargeted metabolomics of point mutants with phospho IN or OUT modifications has the potential to address this functionality problem. From phosphosite mutants of 26 proteins involved in metabolism, TOR and HOG signaling as a proof-of-concept, we were able to recover 10 of the 13 positive controls by metabolomics, compared to only three when relying on quantitative growth assays. Importantly, metabolomics was able to detect also more subtle metabolic effects that were phenotypically silent. The other three known phosphoproteins were presumably not detected because we did not mutate all relevant phosphosites (Ste50 OUT) or did not chose the appropriate conditions that required activity of these proteins (Crz1 OUT and Put3 OUT) [18, 37, 38].

Of the remaining 13 so far uncharacterized phosphosites, metabolomics provides evidence that those on Cys3, Gln3, Gut1, Nbp2, Pfk2, Tco89 and Tip41 are generally functional. At least Gln3 and Tip41 had been suspected to be regulated by phosphorylation, although no phosphosites were identified so far [46–48]. The strongest evidence in our data was for S79 on Tip41, an essential regulatory protein in TOR signaling whose role upon activation of TOR is not yet clearly established [29, 49]. The exclusively strong effect of Tip41 OUT during growth on a poor nitrogen source suggests that S79 is phosphorylated when the TOR complex is inactive and becomes dephosphorylated upon activation of TOR. Since our recovery of positive controls was fairly good, we believe that the newly tested phosphosites for which we could not obtain functionality evidence are indeed mostly without functional relevance. The employed, metabolomics-based scoring system allowed classification of functionality, but does not directly identify the regulatory consequences of phosphorylation. Nevertheless, in several cases the metabolite profiles provided starting hypotheses for the specific biological function without prior knowledge. For instance accumulation of pathway end products suggested that phosphorylation of Lys20, the first enzyme in lysine biosynthesis, specifically regulates lysine pathway flux. The correlation of Pbs2 IN and Hog1 OUT metabolite profiles strongly suggested that the two phosphorylation events have a similar function in mediating HOG signal flow. However, changes in metabolism were not always intuitive and possibly not direct consequences of the mutated phosphosites, as observed in the Gpd1 and Gpd2 mutants. The main benefit of the metabolomics data in the process of understanding phosphosite functionality is to point out relevant cases efficiently, which can then be investigated in more depth by other methods or in combination with proteomics and transcriptomics data.

Even though about half of the tested phosphosites appeared to be functional, most phosphomutants had no detectable growth phenotype and exhibited relatively modest metabolic changes, both in number and magnitude. This apparent resilience to point mutations does not come entirely unexpected given that the much more drastic intervention of 118 kinase and phosphatase deletions in *S. cerevisiae* resulted in few detectable growth phenotypes where only 26% of the mutants exhibited a significant transcriptional response and about 40% had no detectable metabolite response [26, 50]. Several reasons are likely to contribute to this robustness, including the large overlap and redundancy both in kinase targets and phosphosites on a given protein [2, 51–53]. Additionally, adaptation to a new steady state in our experiments will mask many direct regulatory effects and our population-based measurements will have missed, for example, cell cycle specific regulation events.

## Conclusions

In this study, we investigated the potential of untargeted metabolomics along with growth analysis to identify functionality of phosphosites in a higher throughput. While growth analysis was suitable to detect functionality of a small number of phosphosites, metabolomics allowed recovering most of the previously characterized sites, along with some novel functional phosphosites and aided in hypothesis generation on biological roles of functionally relevant sites. Our results demonstrate the potential of untargeted metabolomics to experimentally identify functionality and conditional relevance of post-translational modifications at high throughput to complement current computational approaches [2, 52–55].

## Methods

### Strains used in this study

All phosphosite mutants were generated in *S. cerevisiae* wild-type FY4 using the delitto perfetto method [21], the oligonucleotides used in this study are listed in Additional file 9. Briefly, in a first step, a cassette containing a positive and a negative selection marker for growth on geneticin or galactose containing media was amplified from pCORE-Kp53 using primers with flanking region homologous to the genomic region of the phosphosite of interest. The cassette was inserted via homologous recombination into the region of interest into wild-type FY4. Next, the entire cassette was replaced via homologous recombination with oligonucleotides containing the desired point mutation, resulting in genomic point mutants of candidate phosphosites free of selection markers. Mutants were selected after confirmation of proper integration of the desired mutation by colony PCR and sequencing of the manipulated genomic region +/− 200 base pairs. Up to three independent mutant replicates per phosphosite were generated. Since no differences in the metabolic profiles were observed between replicates of phosphomutants with strong metabolic effects, the genetic approach was considered reliable and data from untargeted metabolomics and growth phenotype analysis of the replicates was merged for analysis.

### Growth phenotype analysis

All strains were grown in phthalate-buffered Verduyn minimal media supplemented with different carbon and nitrogen sources [56]: glucose/NH_4_^+^ media contained 20 g l^−1^ glucose and 5 g l^−1^ (NH_4_)_2_SO_4_, pyruvate/NH_4_^+^ media contained 24.42 g l^−1^ sodium pyruvate and 5 g l^−1^ (NH_4_)_2_SO_4_, glucose/glutamine media contained 20 g l^−1^ glucose, 5 g l^−1^ K_2_SO_4_ and 1 g l^−1^ glutamine and glucose/proline media contained 20 g l^−1^ glucose, 5 g l^−1^ K_2_SO_4_ and 1 g l^−1^ proline.

Precultures with biological triplicates of each strain were grown at 30 °C, 300 rpm, in deep 96-well plates in 1 ml of media, using one glass bead (Ø 4 mm) per well to improve mixing efficiency. The preculture was used for inoculation of microtiter plates at a starting optical density of 595 nm (OD_595_) of 0.03. Growth was monitored via light scattering using the Biolector® plate reader at 30 °C, 1000 rpm. All experiments were run in duplicates. The growth rate for all replicates was determined by a linear fit to the log-transformed OD_595_ growth curve. Maximum cell densities were determined as the end point of growth at the entry of the stationary phase. Clear outliers such as non-growing replicates and single replicates with different growth signal e.g. due to technical reasons were removed manually. A two-sided 2-sample t-test assuming unequal variance was performed to compare the significance of difference in growth rate and maximally reached cell density between mutants and wild-type. The *p*-values were corrected for multiple testing as described by Storey [57]. Growth rates or maximally reached cell densities of at least 10% difference to the wild-type and a *p*-value < 10^−3^ were considered significant. Abnormal lag time and growth curve shape were qualitatively assigned.

### Metabolite extraction, sample measurement and data processing for untargeted metabolomics

Metabolites were extracted from deep 96-well cultures when the average OD_595_ of the plate was around 1 and all cultures had doubled at least twice. The entire plates was centrifuged for 1 min at −9 °C, 4000 rpm. The supernatant was discarded, 150 ul of 80 °C 75% (*v/v*) ethanol in 10 mM ammonium acetate at pH 7.5 was added to each well. For extraction, plates were incubated for 5 min at 80 °C with three vortexing steps. The plates were centrifuged again for 5 min and the supernatant containing the metabolites was transferred to fresh plates for analysis. The metabolite extraction and measurements of extracts were conducted in two batches of mutants (Additional file 1). Mutants were grown in triplicates on the 96-well plate while wild-type was inoculated in four to five replicates per plate. Each culture plate was grown and extracted twice on different days yielding to day replicates for every mutant.

Metabolites were measured by direct flow double injection of extracts on an Agilent 6550 series quadrupole TOF MS with the help of a GERSTEL MPS2. Ions within a mass/charge ratio range of 50–1000 were measured [33]. The ions were annotated either using the genome-wide reconstruction model of *S. cerevisiae* metabolism by Dobson et al. (2010) or, for pathway enrichment analysis, the *S. cerevisiae* reactants defined in the KEGG database [34, 58]. Only ions corresponding to deprotonated metabolites were considered for further analysis. The entire annotation is provided in Additional file 10. All data analysis was conducted using Matlab (The Mathworks, Natick).

Measured ion intensities of samples taken at the exponential phase were corrected for potential signal drifts during the measurement run by applying locally weighted scatter plot smoothing (LOWESS). For further processing and retrieval of changes in metabolites only samples in a restricted OD_595_ range between 0.6 - 1.7, with marginal variation between conditions and sampling batches, were considered. This OD range contained the majority of all exponentially growing samples. A fitted intensity curve was used as OD-wise reference curve to compare each ion of every sample to the average intensity at its specific sampling OD. This OD-wise comparison allows to account for potential impacts of the extraction OD on ion intensity and comparison of samples with different extraction ODs. Fitting was done by smoothing the measured intensities of all samples vs. the OD_595_ of the samples using LOWESS. Including all samples for the calculation of the reference curve enables a thorough covering of the entire OD range and renders the reference curve robust. Further, LOWESS was performed in robust mode to prevent individual strong metabolic changes from having a dominating impact on the reference curve. An adapted procedure for the calculation of the OD-wise reference curve was chosen for batch 2, as the number of Pda1 OUT and Pda1 IN mutant strains strongly dominated this batch, being the only mutants generated in three replicates in batch 2. Therefore, three Pda1 OUT and three Pda1 IN replicates were chosen randomly from each of the two deep 96-well plate replicate for the calculation of the reference curve, thereby adapting the number of replicates for this mutant to the remaining phosphomutants in batch 2. Random sample choice was done 1000 times, and the final reference curve for batch 2 was calculated as the median of the references curves of all permutations.

For every ion we determined the fold change compared to the intensity of the ion-specific reference curve at the corresponding sample-OD. Fold changes for all samples of each mutant were pooled and ion-wise outliers that deviate more than two standard deviations from the mean fold change were excluded. The median log2 fold change was determined and a *p*-value was assigned by comparing the fold changes of one mutant to all other samples using a two-sided 2-sample t-test assuming unequal variance (Additional files 7 and 8). The *p*-values were corrected for multiple testing as described before [57]. A cutoff on the fold change was applied for removal of very small changes. For this purpose, the absolute log2 fold changes of all wild-type ions were pooled, and the 97.5% quantile was determined to be at 0.381. Accordingly, corresponding to a 30% change, the log2 fold change cutoff was defined at 0.3785. Ions with a log2 fold change stronger than +/−0.3785 and a corrected *p*-value < 10^-3^ were considered significantly changing.

### Analysis of untargeted metabolomics data

Pathway enrichment analysis was performed based on the approach detailed in Kühne et al. (2015) [59]. Briefly, metabolites passing a relaxed cutoff (*p*-value 0.1 and absolute log2 fold change of 0.1375, corresponding to a 10% change) were sorted according to their absolute change, and a hypergeometric test was performed to calculate the enrichment of each metabolic pathway. For the enrichment calculation, the hit subset was defined starting with only the top hit of the generated list of sorted metabolites. For each subsequent enrichment analysis, the hit subset was extended by adding the next metabolite from the sorted list. Eventually the most significant result of all generated enrichments based on the different hit subsets was considered. Pathway definition was retrieved from KEGG [58]. Ions that were annotated to several metabolites within a given metabolic pathway were lumped to one entry.

To retrieve the mutant-pair correlation over all conditions, a pair-wise Pearson correlation coefficient was calculated for all mutant-pairs for each condition. Calculation was based on log2 fold changes of ions corresponding to deprotonated metabolites. The correlation coefficients of all mutant-pairs showed a normal distribution that centered around 0 (Additional file 2: Figure S5A). The four correlation coefficients from each mutant-pair were compared using a 2-sample t-test against this distribution of correlation coefficients of all remaining mutant-pairs in order to find meaningful mutant-pair correlations. Mutant-pairs with a *p*-value below 0.01 were considered as correlating significantly with each other throughout all conditions. For analyzing self-correlation of mutants across conditions, the pair-wise Pearson correlation coefficient was calculated for each mutant for every condition-pair combination, resulting in six correlation coefficients per phosphomutant. Calculation was based on log2 fold changes of ions corresponding to deprotonated metabolites. The six correlation coefficients of each mutant were compared against an average of zero performing a t-test. Mutants with a *p*-value below 0.01 were considered as correlating significantly with themselves across conditions.

### Targeted LC-MS/MS analysis

For targeted LC-MS/MS analysis, 500 ml shake flasks containing 50 ml glucose/NH_4_^+^ medium were inoculated with overnight precultures grown in the same medium. At OD_595_ 1 +/− 0.15, 1 ml of culture was quenched by mixing with 4 ml 60% (*v/v*) methanol precooled at -40 °C. 100 ul uniformly-labeled ^13^C yeast extract was added as internal standard and metabolites from cell pellets were extracted by incubation with 1 ml 75% (*v/v*) 80 °C ethanol for 3 min, with three vortexing steps. Samples were dried and resuspended in MilliQ water and analyzed for quantitative targeted analysis of specific metabolites as described before with the exception that citrulline was normalized to the ^13^C-arginine signal for lack of ^13^C-citrulline signal in the standard [60]. Mean and standard deviation of three wild-type and four Gpd1 OUT replicates was determined.

### Spotting assay

Yeast cells from SC-galactose plates were dissolved in 0.9% (*w/v*) NaCl and OD_595_ was adjusted to 1. Serial 1:10 dilutions were prepared and 3.5 ul were spotted onto SC-glucose plates with or without 0.5 M NaCl. Plates without NaCl were incubated at 16 °C, 30 °C or 42 °C, while plates with NaCl were incubated at 30 °C for 2–4 days.

### Hog1 phosphorylation analysis in the Pbs2 mutants

Yeast strains were grown in YPD at 30 °C to an OD_660_ of 0.6. Cells were stressed with NaCl (final concentration 0.4 M) and samples were taken at the indicated time points. Proteins were extracted by glass bead lysis, separated on a 10% (*w/v*) SDS page and transferred to a PVDF membrane. The membrane was probed with anti-ph-p38 (Cell Signaling 9215 L) and anti-total Hog1 (Santa Cruz Biotechnology sc6815).

## Additional files

Additional file 1: Additional information on mutated phosphosites. (XLSX 17 kb)
Additional file 2: Additional figures S1 – S7 and additional table S1 – S2. (DOCX 1049 kb)
Additional file 3: Results on growth rates and maximal cell densities from growth experiments. (XLSX 30 kb)
Additional file 4: Results from pathway enrichment analysis. (XLSX 69 kb)
Additional file 5: Results correlation analysis phosphomutants within one condition. (XLSX 70 kb)
Additional file 6: Results self-correlation analysis phosphomutants across condition. (XLSX 14 kb)
Additional file 7: Log 2 fold changes from untargeted metabolomics. (XLSX 1220 kb)
Additional file 8: Corrected *p*-values from untargeted metabolomics. (XLSX 1258 kb)
Additional file 9: List of oligonucleotides used in this study. (XLSX 17 kb)
Additional file 10: Detailed annotation from untargeted metabolomics. (XLSX 303 kb)

## Abbreviations

LOWESS: Locally weighted scatter plot smoothing
Phosphosite: Phosphorylation site
PTM: Post-translational modification
TOF-MS: Time of Flight mass spectrometer

## References

[1] Cherry JM, Hong EL, Amundsen C, Balakrishnan R, Binkley G, Chan ET (2012). Saccharomyces genome database: the genomics resource of budding yeast. Nucleic Acids Res.

[2] Beltrao P, Bork P, Krogan NJ, van Noort V (2013). Evolution and functional cross-talk of protein post-translational modifications. Mol Syst Biol.

[3] Choudhary C, Mann M (2010). Decoding signalling networks by mass spectrometry-based proteomics. Nat Rev Mol Cell Biol.

[4] Bodenmiller B, Wanka S, Kraft C, Urban J, Campbell D, Pedrioli PG (2010). Phosphoproteomic analysis reveals interconnected system-wide responses to perturbations of kinases and phosphatases in yeast. Sci Signal.

[5] Jünger MA, Aebersold R (2014). Mass spectrometry-driven phosphoproteomics: Patterning the systems biology mosaic. Wiley Interdiscip Rev Dev Biol.

[6] Landry CR, Levy ED, Michnick SW (2009). Weak functional constraints on phosphoproteomes. Trends Genet.

[7] Levy ED, Michnick SW, Landry CR (2012). Protein abundance is key to distinguish promiscuous from functional phosphorylation based on evolutionary information. Philos Trans R Soc B Biol Sci.

[8] Oliveira AP, Sauer U (2012). The importance of post-translational modifications in regulating Saccharomyces cerevisiae metabolism. FEMS Yeast Res.

[9] Mok J, Kim PM, Lam HYK, Piccirillo S, Zhou X, Jeschke GR (2010). Deciphering protein kinase specificity through large-scale analysis of yeast phosphorylation site motifs. Sci Signal.

[10] Ellis JJ, Kobe B (2011). Predicting protein kinase specificity: Predikin update and performance in the DREAM4 challenge. PLoS One.

[11] Oliveira AP, Ludwig C, Picotti P, Kogadeeva M, Aebersold R, Sauer U (2012). Regulation of yeast central metabolism by enzyme phosphorylation. Mol Syst Biol.

[12] Kanshin E, Bergeron-Sandoval L-P, Isik SS, Thibault P, Michnick SW (2015). A cell-signaling network temporally resolves specific versus promiscuous phosphorylation. Cell Rep.

[13] Rohde JR, Trinh J, Sadowski I (2000). Multiple signals regulate GAL transcription in yeast. Mol Cell Biol.

[14] Wu C, Arcand M, Jansen G, Zhong M, Iouk T, Thomas DY (2003). Phosphorylation of the MAPKKK regulator Ste50p in Saccharomyces cerevisiae: a Casein kinase I phosphorylation site is required for proper mating function. Eukaryot Cell.

[15] Gopalbhai K, Jansen G, Beauregard G, Whiteway M, Dumas F, Wu C (2003). Negative regulation of MAPKK by phosphorylation of a conserved serine residue equivalent to Ser212 of MEK1. J Biol Chem.

[16] Park TS, O’Brien DJ, Carman GM (2003). Phosphorylation of CTP synthetase on Ser36, Ser330, Ser354, and Ser454 regulates the levels of CTP and phosphatidylcholine synthesis in Saccharomyces cerevisiae. J Biol Chem.

[17] Mollapour M, Piper PW (2007). Hog1 mitogen-activated protein kinase phosphorylation targets the yeast Fps1 aquaglyceroporin for endocytosis, thereby rendering cells resistant to acetic acid. Mol Cell Biol.

[18] Leverentz MK, Campbell RN, Connolly Y, Whetton AD, Reece RJ (2009). Mutation of a phosphorylatable residue in Put3p affects the magnitude of rapamycin-induced PUT1 activation in a Gat1p-dependent manner. J Biol Chem.

[19] Pereira J, Pimentel C, Amaral C, Menezes RA, Rodrigues-pousada C (2009). Yap4 PKA- and GSK3-dependent phosphorylation affects its stability but not its nuclear localization. Yeast.

[20] Lee YJ, Jeschke GR, Roelants FM, Thorner J, Turk BE (2012). Reciprocal phosphorylation of yeast glycerol-3-phosphate dehydrogenases in adaptation to distinct types of stress. Mol Cell Biol.

[21] Storici F, Resnick MA (2006). The delitto perfetto approach to in vivo site-directed mutagenesis and chromosome rearrangements with synthetic oligonucleotides in yeast. Methods Enzymol.

[22] Wang HH, Isaacs FJ, Carr PA, Sun ZZ, Xu G, Forest CR (2009). Programming cells by multiplex genome engineering and accelerated evolution. Nature.

[23] Dicarlo JE, Conley AJ, Penttilä M, Jäntti J, Wang HH, Church GM (2013). Yeast oligo-mediated genome engineering (YOGE). ACS Synth Biol.

[24] Mans R, van Rossum HM, Wijsman M, Backx A, Kuijpers NGA, van den Broek M (2015). CRISPR/Cas9: a molecular Swiss army knife for simultaneous introduction of multiple genetic modifications in Saccharomyces cerevisiae. FEMS Yeast Res.

[25] Mathew AK, Padmanaban VC (2013). Metabolomics: the apogee of the omics trilogy. Int J Pharm Pharm Sci.

[26] Schulz JC, Zampieri M, Wanka S, Von Mering C, Sauer U (2014). Large-scale functional analysis of the roles of phosphorylation in yeast metabolic pathways. Sci Signal.

[27] Oliveira AP, Ludwig C, Zampieri M, Weisser H (2015). Dynamic phosphoproteomics reveals TORC1-dependent regulation of yeast nucleotide and amino acid biosynthesis. Sci Signal.

[28] Saito H, Posas F (2012). Response to hyperosmotic stress. Genetics.

[29] Loewith R, Hall MN (2011). Target of rapamycin (TOR) in nutrient signaling and growth control. Genetics.

[30] Bodenmiller B, Campbell D, Gerrits B, Lam H, Jovanovic M, Picotti P (2008). PhosphoPep-a database of protein phosphorylation sites in model organisms. Nat Biotechnol.

[31] Schüller C, Brewster JL, Alexander MR, Gustin MC, Ruis H (1994). The HOG pathway controls osmotic regulation of transcription via the stress response element (STRE) of the Saccharomyces cerevisiae CTT1 gene. EMBO J.

[32] Maeda T, Wurgler-Murphy SM, Saito H (1994). A two-component system that regulates an osmosensing MAP kinase cascade in yeast. Nature.

[33] Fuhrer T, Heer D, Begemann B, Zamboni N (2011). High-throughput, accurate mass metabolome profiling of cellular extracts by flow injection-time-of-flight mass spectrometry. Anal Chem.

[34] Dobson PD, Smallbone K, Jameson D, Simeonidis E, Lanthaler K, Pir P (2010). Further developments towards a genome-scale metabolic model of yeast. BMC Syst Biol.

[35] Ljungdahl PO, Daignan-Fornier B (2012). Regulation of amino acid, nucleotide, and phosphate metabolism in Saccharomyces cerevisiae. Genetics.

[36] Macia J, Regot S, Peeters T, Conde N, Solé R, Posas F (2009). Dynamic signaling in the Hog1 MAPK pathway relies on high basal signal transduction. Sci Signal.

[37] Hao N, Zeng Y, Elston TC, Dohlman HG (2008). Control of MAPK specificity by feedback phosphorylation of shared adaptor protein Ste50. J Biol Chem.

[38] Kafadar KA, Cyert MS (2004). Integration of stress responses: modulation of calcineurin signaling in saccharomyces cerevisiae by protein kinase A. Eukaryot Cell.

[39] Maeda T, Takekawa M, Saito H (1995). Activation of yeast PBS2 MAPKK by MAPKKKs or by binding of an SH3-containing osmosensor. Science.

[40] Rodríguez-Peña JM, García R, Nombela C, Arroyo J (2010). The high-osmolarity glycerol (HOG) and cell wall integrity (CWI) signalling pathways interplay: a yeast dialogue between MAPK routes. Yeast.

[41] Bermejo C, Garcı R, Rodrı JM, Concepcio D, Posas F, Arroyo J (2008). The sequential activation of the yeast HOG and SLT2 pathways is required for cell survival to cell wall stress. Mol Biol Cell.

[42] Ansell R, Granath K, Hohmann S, Thevelein JM, Adler L (1997). The two isoenzymes for yeast NAD + − dependent glycerol 3-phosphate dehydrogenase encoded by GPD1 and GPD2 have distinct roles in osmoadaptation and redox regulation osmoadaptation. EMBO J.

[43] Nissen TL, Hamann CW, Kielland-Brandt MC, Nielsen J, Villadsen J (2000). Anaerobic and aerobic batch cultivations of Saccharomyces cerevisiae mutants impaired in glycerol synthesis. Yeast.

[44] Melamed D, Pnueli L, Arava Y (2008). Yeast translational response to high salinity: global analysis reveals regulation at multiple levels. RNA.

[45] Bodenmiller B, Aebersold R (2010). Quantitative analysis of protein phosphorylation on a system-wide scale by mass spectrometry-based proteomics. Methods Enzymol.

[46] González A, Ruiz A, Casamayor A, Ariño J (2009). Normal function of the yeast TOR pathway requires the type 2C protein phosphatase Ptc1. Mol Cell Biol.

[47] Kulkarni A, Buford TD, Rai R, Cooper TG (2006). Differing responses of Gat1 and Gln3 phosphorylation and localization to rapamycin and methionine sulfoximine treatment in Saccharomyces cerevisiae. FEMS Yeast Res.

[48] Jacinto E, Guo B, Arndt KT, Schmelzle T, Hall MN (2001). TIP41 interacts with TAP42 and negatively regulates the TOR signaling pathway. Mol Cell.

[49] Oler AJ, Cairns BR (2012). PP4 dephosphorylates Maf1 to couple multiple stress conditions to RNA polymerase III repression. EMBO J.

[50] Van Wageningen S, Kemmeren P, Lijnzaad P, Margaritis T, Benschop JJ, De Castro IJ (2010). Functional overlap and regulatory links shape genetic interactions between signaling pathways. Cell.

[51] Salazar C, Höfer T (2009). Multisite protein phosphorylation - from molecular mechanisms to kinetic models. FEBS J.

[52] Landry CR, Freschi L, Zarin T, Moses AM (2014). Turnover of protein phosphorylation evolving under stabilizing selection. Front Genet.

[53] Minguez P, Parca L, Diella F, Mende DR, Kumar R, Helmer-Citterich M (2012). Deciphering a global network of functionally associated post-translational modifications. Mol Syst Biol.

[54] Beltrao P, Albanèse V, Kenner LR, Swaney DL, Burlingame A, Villén J (2012). Systematic functional prioritization of protein posttranslational modifications. Cell.

[55] Amoutzias GD, He Y, Lilley KS, Van de Peer Y, Oliver SG (2012). Evaluation and properties of the budding yeast phosphoproteome. Mol Cell Proteomics.

[56] Verduyn C, Postma E, Scheffers W a, Van Dijken JP (1992). Effect of benzoic acid on metabolic fluxes in yeasts: a continuous-culture study on the regulation of respiration and alcoholic fermentation. Yeast.

[57] Storey JD (1995). A direct approach to false discovery rates. J R Stat B.

[58] Kanehisa M, Goto S, Sato Y, Kawashima M, Furumichi M, Tanabe M (2014). Data, information, knowledge and principle: back to metabolism in KEGG. Nucleic Acids Res.

[59] Kuehne A, Emmert H, Soehle J, Winnefeld M, Fischer F, Wenck H (2015). Acute activation of oxidative pentose phosphate pathway as first-line response to oxidative stress in human skin cells. Mol Cell.

[60] Buescher JM, Moco S, Sauer U, Zamboni N (2010). Ultrahigh performance liquid chromatography-tandem mass spectrometry method for fast and robust quantification of anionic and aromatic metabolites. Anal Chem.

[61] Stathopoulos-Gerontides A, Guo JJ, Cyert MS (1999). Yeast calcineurin regulates nuclear localization of the Crz1p transcription factor through dephosphorylation. Genes Dev.

[62] Ferrigno P, Posas F, Koepp D, Saito H, Silver PA (1998). Regulated nucleo/cytoplasmic exchange of HOG1 MAPK requires the importin ?? homologs NMD5 and XPO1. EMBO J.

[63] Uhlinger DJ, Yang CY, Reed LJ (1986). Phosphorylation-dephosphorylation of pyruvate dehydrogenase from bakers’ yeast. Biochemistry.

[64] Wanke V, Cameroni E, Uotila A, Piccolis M, Urban J, Loewith R (2008). Caffeine extends yeast lifespan by targeting TORC1. Mol Microbiol.

[65] Proft M, Pascual-Ahuir A, de Nadal E, Ariño J, Serrano R, Posas F (2001). Regulation of the Sko1 transcriptional repressor by the Hog1 MAP kinase in response to osmotic stress. EMBO J.

[66] De Nadal E, Casadomé L, Posas F (2003). Targeting the MEF2-like transcription factor Smp1 by the stress-activated Hog1 mitogen-activated protein kinase targeting the MEF2-like transcription factor Smp1 by the stress-activated Hog1 mitogen-activated protein kinase. Mol Cell Biol.

