# Supplementary material for: Untargeted metabolomics unravels functionalities of phosphorylation sites in Saccharomyces cerevisiae
Source: BMC Syst Biol. 2016 Nov 15;10:104. doi: 10.1186/s12918-016-0350-8 (PMC5109706; doi:10.1186/s12918-016-0350-8)
Supplement: Additional file 2: — Additional figures S1 – S7 and additional table S1 – S2. (DOCX 1049 kb) [file 12918_2016_350_MOESM2_ESM.docx]

**Supplementary**

**Figures**

Figure S1: Example of genetic replicate comparison for a phosphomutant with strong metabolic impact.

Log2 fold changes of all ions annotated as deprotonated metabolites for all independently generated mutant strains for Pda1 OUT in glucose / NH4+ media. Two example metabolites are indicated.

Figure S2: Distribution of log 2 fold changes and cutoff determination.

A: Distribution of absolute log2 fold changes of wild-type across all conditions. 95%, 97.5% and 99% quantiles are indicated. B: Distribution of absolute log2 fold changes across all mutants and conditions that pass the p‑value cutoff of 10^-3^. Ions that do not pass the log2 fold-change cutoff of +/-0.3875 are highlighted with a red shadow.

Figure S3: Coverage of reactants from untargeted metabolomics experiments.

Number of detected reactants of all mutated metabolic enzymes across all conditions using untargeted metabolomics. The number before the slash represents the measured metabolites, the number after the slash is the theoretical number of reactants for any given enzyme based on the genome-wide model of Dobson *et al.* (2010).

Figure S4: Distribution of effects across metabolic pathways.

Distribution across metabolic pathways as defined by KEGG of the average number of significantly changing metabolites (absolute log 2 fold-change > 0.3875 and p‑value < 10^-3^) per phosphomutant. The phosphomutants are divided according to their biological subgroups. The number of changes is normalized by the number of strains and conditions for each of the three groups.

Figure S5: Distribution of the correlation coefficients.

A: Distribution of the correlation coefficients from the correlation analysis of metabolic profiles between all possible mutant pairs for all conditions. B: Distribution of the correlation coefficients from self-correlation analysis where metabolic profiles of mutants were correlated to profiles of the same mutant across different conditions.

Figure S6: Quantification of peptides for Pfk2 phosphomutants.

Relative peptide quantification of Pfk2 phosphomutants and wild-type from this study and from Oliveira *et al.* (2012) growing in ethanol / NH_4_^+^ was determined by SRM. Measured peptide sequences are listed in Additional file 1, Table S2. A: Relative quantification of peptides corresponding to the phosphorylated (NAVSTKPTPPPAPEASAES[phospho]GLSSK) and the non-phosphorylated (NAVSTKPTPPPAPEASA ESGLSSK) isoforms of residue S163. These peptides are not detected in any of the Pfk2 phosphomutants as serine at position 163 is replaced by alanine (Pfk2 OUT strains, indicated in purple and blue). B: Relative quantification of two representative peptides for the determination of the relative abundance of the total Pfk2 protein, showing a ca. 4-fold increased protein concentration in the Pfk2 OUT strain of Oliveira *et al*. C: Relative quantification of various control proteins for verification of consistency of protein levels between strains.

Figure S7: Simplified model of the phosphoregulation of Pbs2 based on Gopalbhai *et al.* (2003).

Pbs2 is regulated by phosphorylation of residues 514, 518 and 508. It is activated by phosphorylation upon osmotic stress at position 514 and 518 either in a SLN1 or a SHO1 branch-dependent manner. Active Pbs2 in turn phosphorylates the Hog1 kinase at position 174 and 176. During adaptation to osmotic stress, Pbs2 is being phosphorylated on a third position 508, which leads to inactivation of the Pbs2 kinase.

Figure S1


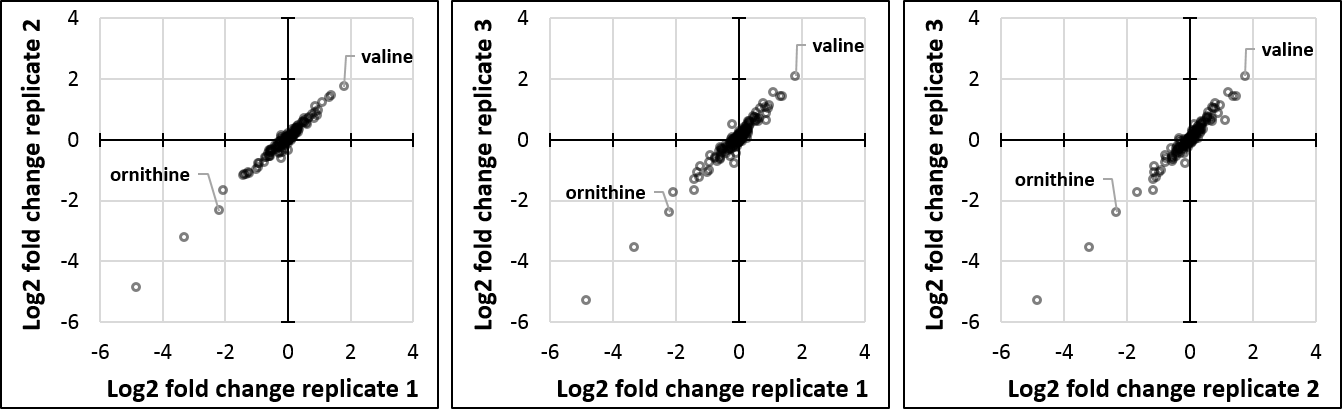


Figure S2


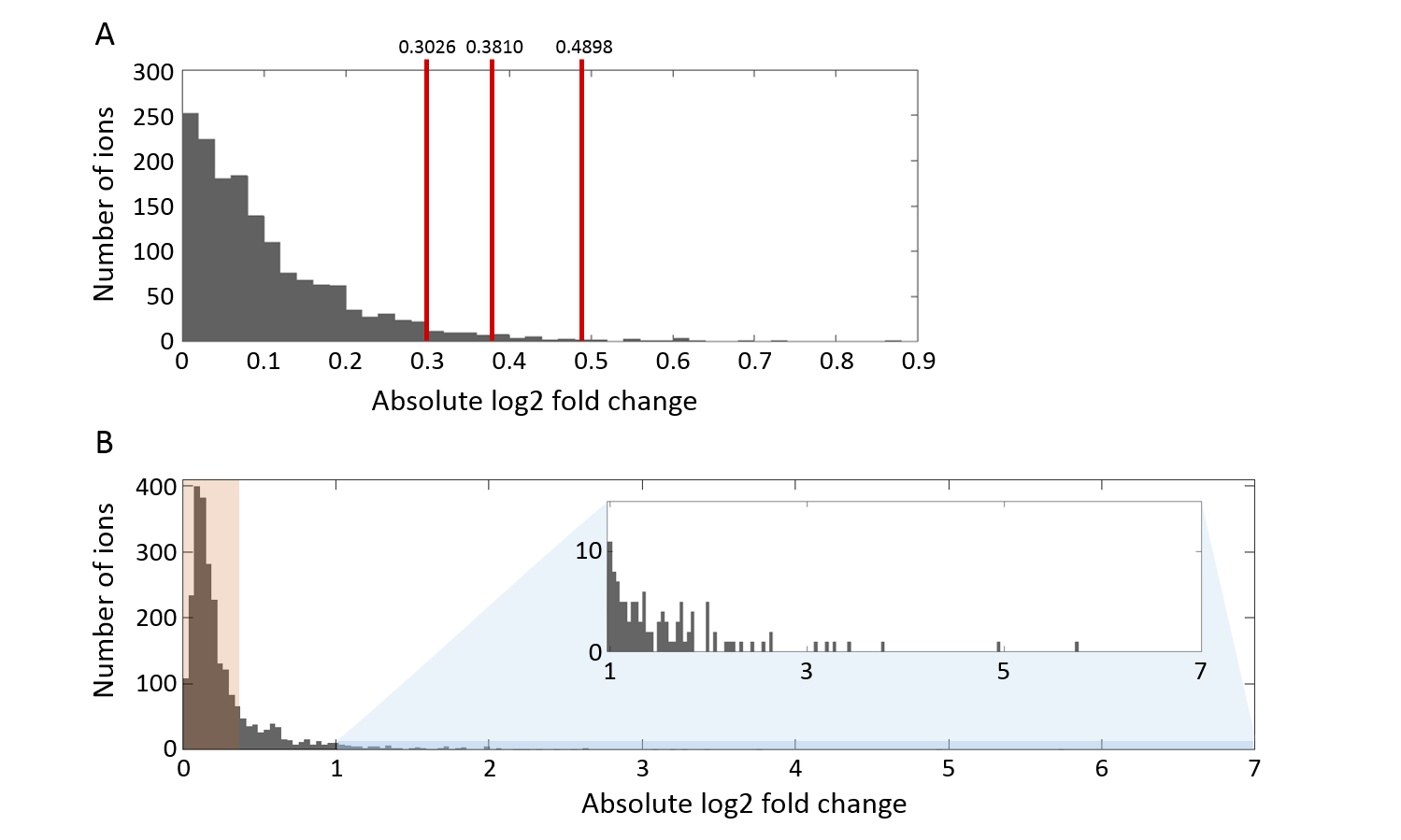


Figure S3


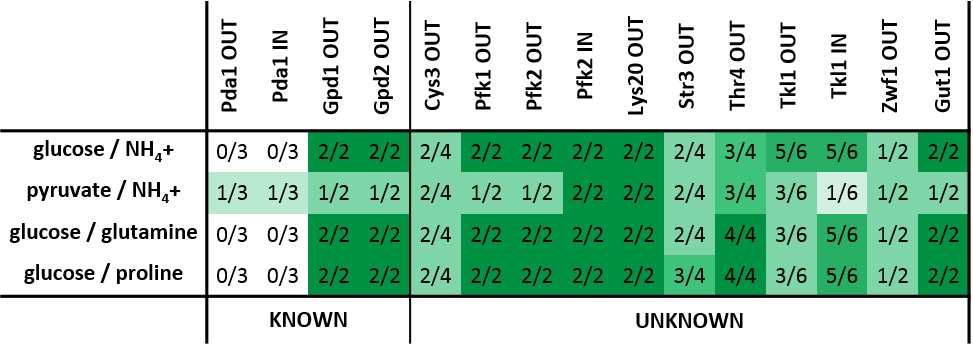


Figure S4
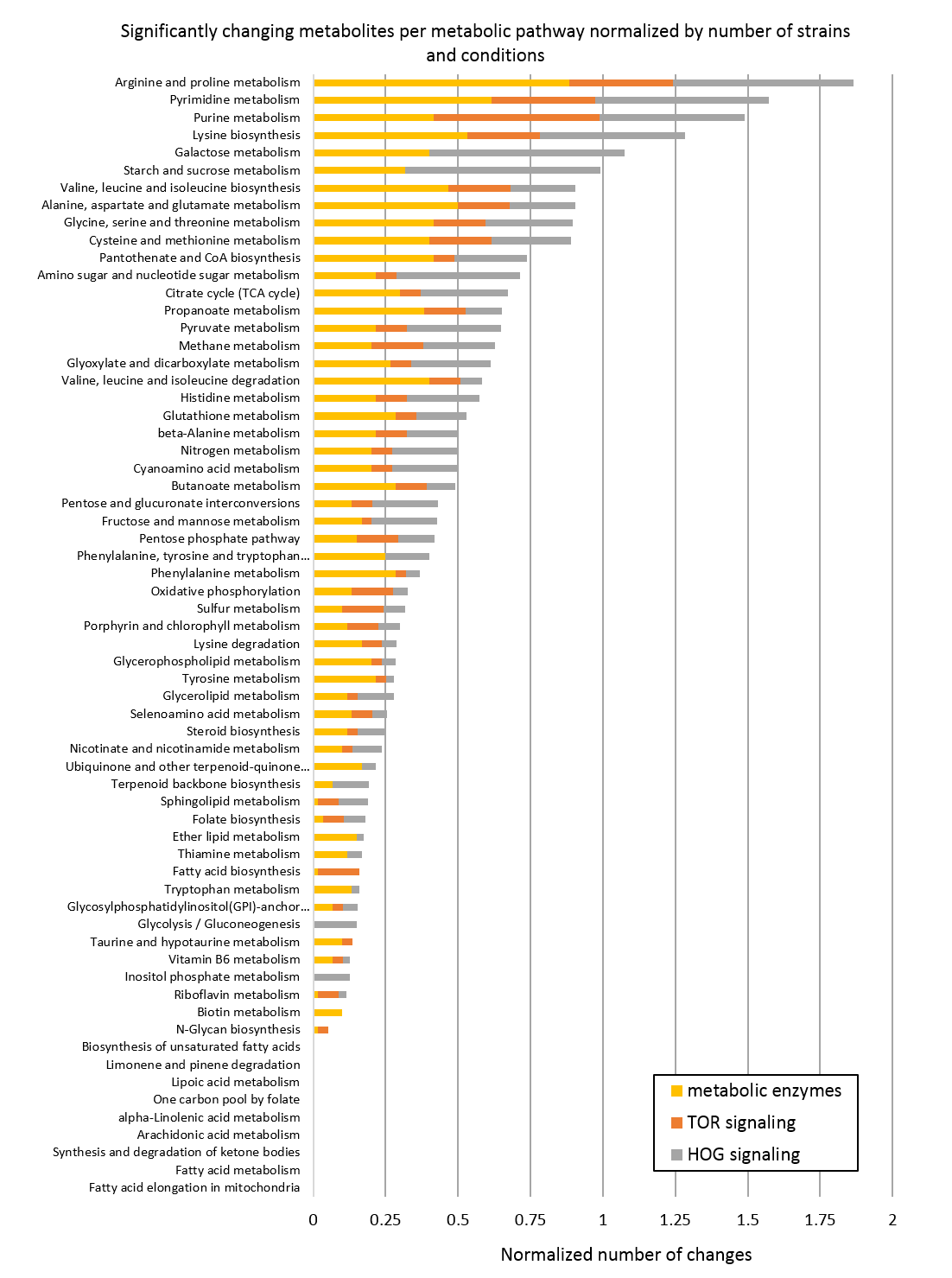


Figure S5


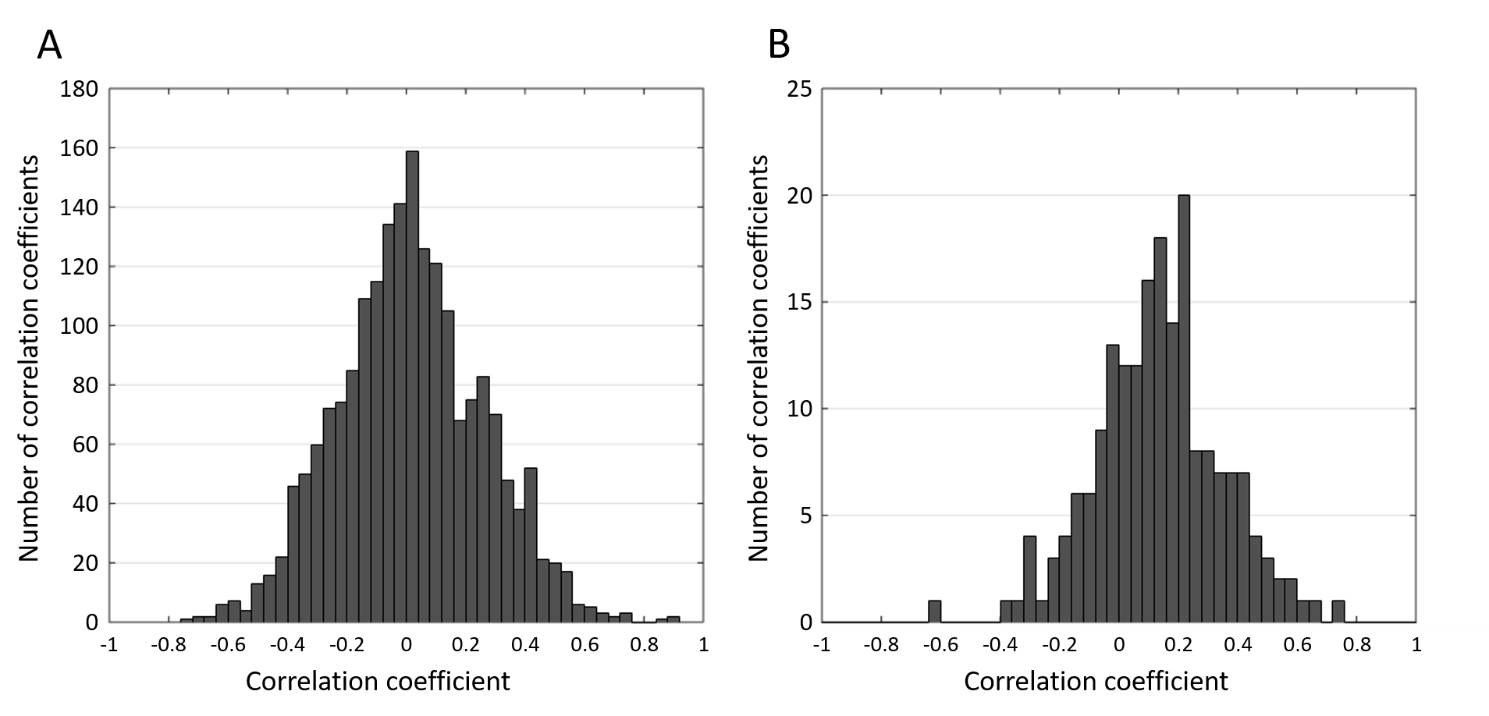


Figure S6
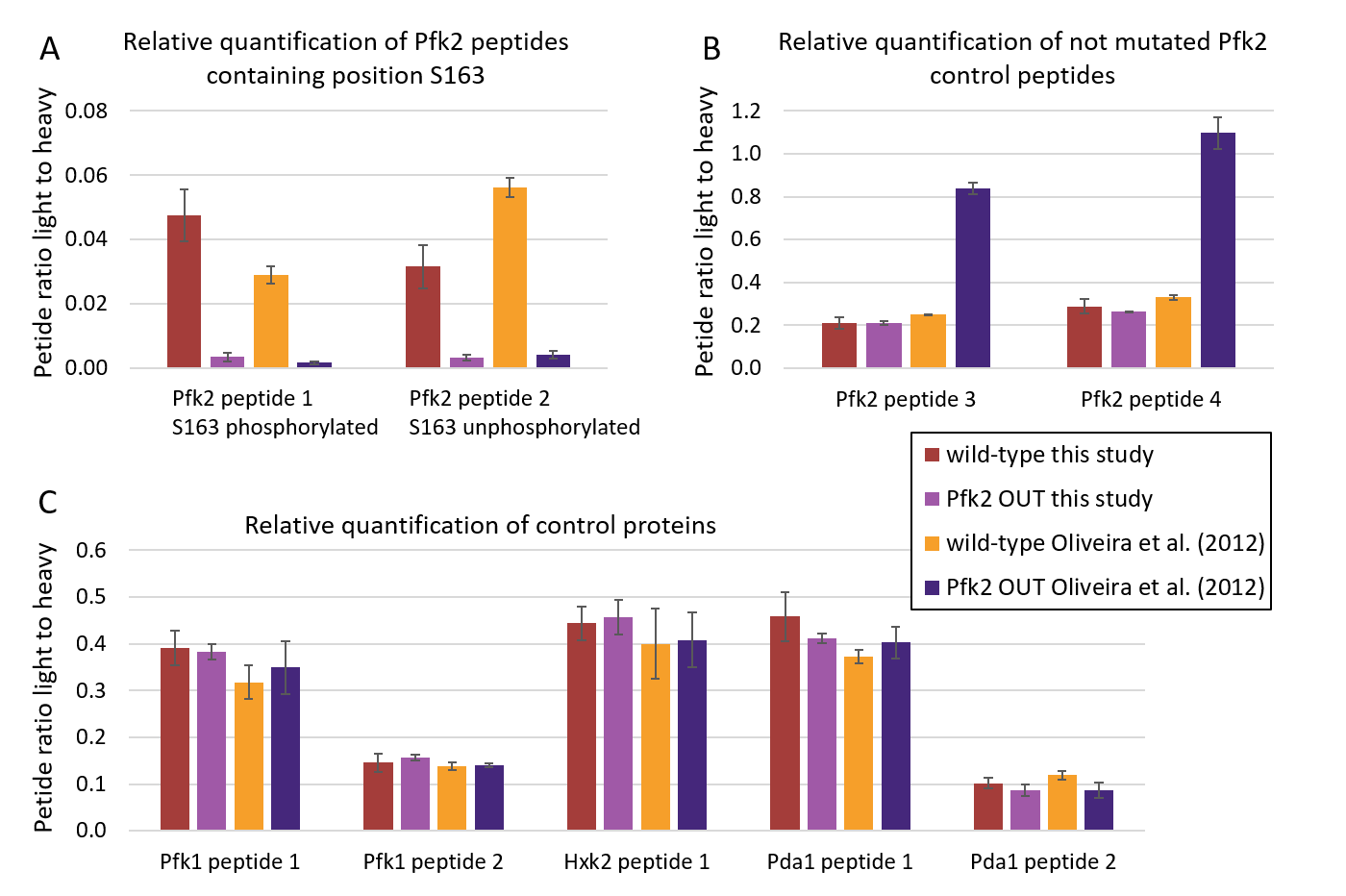


Figure S7


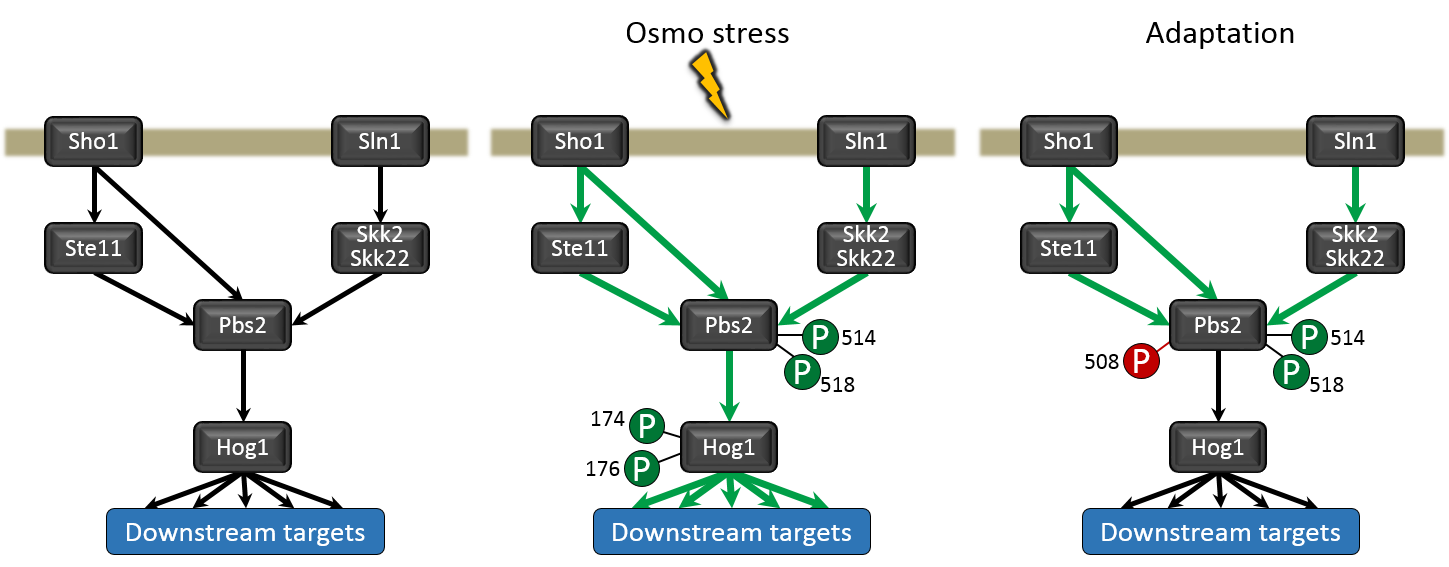


**Tables**

Table S1: Number of annotated ions and metabolites across all conditions and mutant batches.

|  | **glucose / NH_4_^+^ batch 1** | **glucose / NH_4_^+^**  **batch 2** | **pyruvate / NH_4_^+^**  **batch 1** | **pyruvate / NH_4_^+^**  **batch 2** | **glucose / glutamine**  **batch 1** | **glucose / glutamine**  **batch 2** | **glucose / proline**  **batch 1** | **glucose / proline**  **batch 2** |
| --- | --- | --- | --- | --- | --- | --- | --- | --- |
| Detected ions | 7367 | 1705 | 5401 | 2116 | 8092 | 2074 | 7697 | 1953 |
| Ions matching genome-wide model | 247 | 157 | 218 | 122 | 256 | 163 | 259 | 153 |
| Metabolites annotated with genome-wide model | 334 | 224 | 297 | 183 | 338 | 230 | 342 | 225 |
| Ions matching KEGG reaction pairs | 303 | 167 | 283 | 137 | 309 | 177 | 309 | 161 |
| Metabolites annotated with KEGG reaction pairs | 438 | 265 | 398 | 221 | 437 | 279 | 441 | 263 |

Table S2: Peptide sequences measured by SRM for Pfk2 phosphomutants and wild-type.

| Pfk2 peptide 1 | NAVSTKPTPPPAPEASAES[+80]GLSSK |
| --- | --- |
| Pfk2 peptide 2 | NAVSTKPTPPPAPEASAESGLSSK |
| Pfk2 peptide 3 | GWSAEGGTNIGTAR |
| Pfk2 peptide 4 | AIDYVEATANSHSR |
| Pfk1 peptide 1 | AVLEFTPETPSPLIGILENK |
| Pfk1 peptide 2 | AEVAALAAENK |
| Hxk2 peptide 1 | TTQNPDELWEFIADSLK |
| Pda1 peptide 1 | YGGHSMSDPGTTYR |
| Pda1 peptide 2 | YGGHS[+80]MSDPGTTYR |

**Supplementary methods**

**Cultivation conditions and sampling for selected reaction monitoring (SRM)**

500 ml shake flasks containing 50 ml Verduyn minimal media supplemented with 20 ml/L ethanol as carbon source were inoculated with precultures grown in the same medium [1]. At OD_600_ of ca. 0.8, 40 mL of cell culture was sampled with 6.25% (w/v) trichloroacetic acid. Samples were cooled on ice for 10 minutes, centrifuged at 4°C for 5 minutes and washed twice with 10 mL pre-cooled (-20°C) acetone. The cell pellets were frozen in liquid nitrogen and stored at -80 °C until further usage. Three independent biological replicates per strain were prepared.

**Sample processing for SRM measurements**

Yeast samples were thawed on ice and the cell pellets were re-suspended in lysis buffer (8 M urea, 100 mM NH_3_HCO_3_ pH 8.0, 1 mM TCEP). Cell suspensions were disrupted by three 10-min cycles of beads beating at 4°C using glass beads with a diameter of 0.5 mm. After each cycle, lysates were centrifuged for 10 min at 16,000 g and fresh lysis buffer was added. Protein lysates were combined and protein concentrations were determined using a BCA assay according to the manufacturer’s protocol (Thermo Fisher Scientific #23227). From each sample only 100 µg of protein was used for subsequent steps. Protein disulfide bonds were reduced by adding 5 mM tris(2-carboxyethyl)phosphine (TCEP) and incubating for 30 min at 30°C. Free cysteine residues were alkylated with 40 mM iodoacetamide and incubating for 60 min in the dark at room temperature. Subsequently, samples were diluted with 0.05 M ammonium bicarbonate buffer to reach a urea concentration of <2 and 2 µg of sequencing-grade modified trypsin (#608-274-4330, Promega) was added to each sample (protein:trypsin = 50:1). The samples were incubated over night at 30°C with gentle shaking at 300 rpm. At this step nine isotopically labelled synthetic reference peptides (AQUA QuantPro, Thermo Fisher Scientific) for quantification of the 4 target proteins (Pfk2, Pfk1, Hxk2 and Pda1) and two phosphorylation states (Pfk2 and Pda1) were added to all samples in the same concentration, which was chosen to be similar to the endogenous peptides level. To stop the tryptic digest the pH was lowered to pH 2 using trifluoro acetic acid (final concentration 1%). The digested peptide mixtures were purified using C18 cartridges (Sep-Pak tC18, Waters), eluted with 40% (v/v) ACN/0.1% (v/v) TFA, dried in a vacuum centrifuge and re-solubilized in 48 µl 2% (v/v) ACN/0.1% (v/v) FA and 2 µl iRT peptide mix (RT-kit WR, Biognosys, used for retention time scheduling) to a final concentration of 2 mg/ml.

**SRM measurement setup**

SRM measurements were performed on a TSQ Vantage triple quadruple mass spectrometer (Thermo Fischer Scientific) equipped with a nano-electrospray ion source. Peptide separation was carried out on a nano-LC system (Eksigent) with a linear 40 min gradient from 2 to 35% solvent B (solvent A: 98% water, 2% acetonitrile, 0.1% formic acid; solvent B: 98% acetonitrile, 2% water, 0.1% formic acid) at a flow rate of 300 nL/min. An injection volume of 1 µl (2 µg total peptide amount) was loaded onto a 75 µm x 10.5 cm fused silica microcapillary reverse phase column, in-house packed with Magic C18 AQ material (200Å pore, 5 µm diameter, Michrom Bioresources) for each sample. The mass spectrometer was operated in positive ion mode using electron spray ionization (ESI) with a collision gas pressure of 1.5 mTorr, a capillary temperature of 280°C and a spray voltage of +1200 V. SRM transitions were monitored with a mass window of 0.7 half maximum peak width (unit resolution) in Q1 and Q3. Optimal SRM-assay parameters (most-intense precursor charge state, 4 to 6 most-intense transitions, collision energy and retention time) were obtained by SRM-triggered MS/MS experiments on the applied triple quadrupole instrument. Measurements were carried out in scheduled mode applying a retention time window of 5 min, cycle time of ca. 2 s and a minimal dwell time of approximately 20s.

**SRM data analysis**

SRM-derived transition chromatograms were analyzed using the open source software Skyline [2]. The sum of all transition peak areas monitored for a specific endogenous (light) and standard (heavy) peptide was calculated. From the light versus heavy peptide ratio, the relative peptide abundance in the sample was determined and directly compared between samples.

SRM data can be visualized in and downloaded from Panorama (https://panoramaweb.org) using the following link:

https://panoramaweb.org/labkey/targetedms/Aebersold/ludwig/Raguz_2016_Pfk2/showPrecursorList.view?id=7021

**Supplementary references**

1. Verduyn C, Postma E, Scheffers W a., Van Dijken JP. Effect of benzoic acid on metabolic fluxes in yeasts: A continuous-culture study on the regulation of respiration and alcoholic fermentation. Yeast. 1992;8:501–17.

2. MacLean B, Tomazela DM, Shulman N, Chambers M, Finney GL, Frewen B, et al. Skyline: An open source document editor for creating and analyzing targeted proteomics experiments. Bioinformatics. 2010;26:966–8.
